# Leptospirosis-associated meningitis in a patient with sjögren’s syndrome: a case report

**DOI:** 10.1186/s12879-023-08794-9

**Published:** 2023-11-09

**Authors:** Yifan Zhang, Yong Zheng

**Affiliations:** Neurological Center, Shenzhen Baoan People’s Hospital, Shenzhen, China

**Keywords:** Leptospirosis, Sjögren’s syndrome, Autoimmune Diseases, High-throughput sequencing

## Abstract

**Background:**

Leptospirosis is a zoonotic disease that afflicts both humans and animals. It progresses from flu-like symptoms to more severe hepatic and renal failure, and may also lead to aseptic meningitis. Individuals with autoimmune diseases (ADs) are potentially more susceptible to Leptospirosis. Thus far, limited data has documented the association between Leptospirosis and autoimmune disorders.

**Case presentation:**

The patient had a definitive pathological diagnosis of Sjögren’s syndrome (SS). Due to recurrent headaches, the patient sought consultation with a neurologist. Lumbar puncture revealed elevated white blood cells and protein levels in the cerebrospinal fluid, along with decreased glucose. Tuberculous meningitis was suspected. Radiographic imaging exhibited meningeal enhancement, ventricular enlargement, and hydrocephalus. The patient commenced treatment with anti-tuberculosis therapy and corticosteroids. Subsequently, high-throughput sequencing (HTS) of cerebrospinal fluid identified the presence of *Leptospira interrogans*. The patient was ultimately diagnosed with Leptospiral meningitis, and underwent antimicrobial and immunosuppressive therapy, resulting in stabilization of the condition and gradual symptom recovery.

**Conclusions:**

The case highlights the challenges in diagnosing and managing leptospirosis-related meningitis in the presence of ADs and emphasizes the importance of utilizing HTS for accurate pathogen detection. The potential correlation between leptospirosis and SS warrants further investigation, as does the need for multidisciplinary involvement in treatment strategies for such complex cases.

**Supplementary Information:**

The online version contains supplementary material available at 10.1186/s12879-023-08794-9.

## Background

Leptospirosis is a prevalent zoonotic disease in rural areas worldwide caused by *Leptospira* spp. The genus *Leptospira* is a member of Spirochaetes, characterized by its uniquespiral-shaped form [1]. Globally, the morbidity and mortality rates of leptospirosis in humans are approximated at 1 million and 60,000 cases, respectively [2]. Leptospiral infection could instigate a spectrum of clinical manifestations, extending from mild, non-icteric leptospirosis to a more severe affliction known as Weil’s syndrome. Interestingly, neurological symptoms are not uncommon in patients afflicted with Leptospirosis, with 10–15% of them experiencing such manifestations [3]. The most frequently observed neurological implication is aseptic meningitis.

Leptospirosis is spread through direct contact with infectious animal excretions and body fluids, or through indirect contact with carrier mammals (such as contaminated water or soil) [4]. Rodents are the most common carriers of *Leptospira*. Other predisposing factors include immune-related disorders and immunosuppressive states.

SS, an AD marked by xerophthalmia, xerostomia, and joint pain due to lymphocytic glandular infiltration, is potentially linked to an elevated risk in individuals with Leptospirosis [5]. However, the exact causal relationship and mechanisms remain unclear in existing research.

In this report, we present a case wherein symptomatic aseptic meningitis arose within the context of a diagnosed condition known as SS. The patient presented an absence of jaundice and pyrexia, and did not manifest symptoms of encephalomyelitis or neuromuscular pathology. Remarkably, a fortuitous discovery of the presence of *Leptospira interrogans* was made through next-generation sequencing (NGS) of cerebrospinal fluid (CSF). Ultimately, the patient underwent a course of antibiotics therapy and immunomodulatory treatment, leading to a gradual amelioration of the symptoms.

## Case presentation

**Fig. 1:**
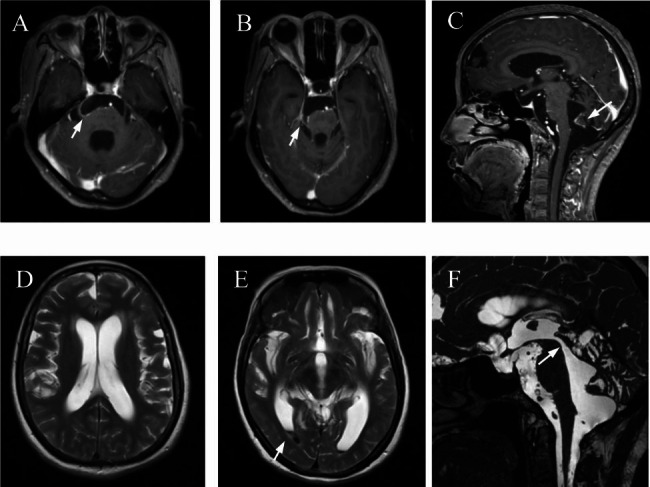
Manifestations of meningitis and hydrocephalus in the patient. **A:** T1-weighted image with contrast enhancement reveals enhanced pia mater in the right cerebellar pontine angle cistern. **B:** T1-weighted image with contrast enhancement shows enhanced pia mater in the right pontine cistern. **C:** T1-weighted image with contrast enhancement in sagittal position reveals enhanced pia mater in the fourth ventricle. **D:** T2-weighted image shows enlarged lateral ventricle. **E:** T2-weighted image shows hemorrhage in the occipital horn of the right lateral ventricle. **F:** T2-weighted image in sagittal position shows unobstructed cerebral aqueduct of midbrain, suggesting communicating hydrocephalus

The patient, a 49-year-old female, has a medical history of rheumatoid arthritis. She has intermittently used steroids and has no personal history of immune deficiency. She has been experiencing headaches for the past three years and was admitted to our hospital. Four years ago, in 2019, she sought medical attention from the neurology department for frontal pain. She did not exhibit symptoms of fever or jaundice. Upon physical examination, there was no significant neck stiffness or signs of meningeal irritation. Cerebrospinal fluid analysis revealed a white blood cell count of 8 [0–5] cells/mm^3^, protein levels of 2938 [150–450] mg/L, and glucose levels of 2.09 [2.5–4.5] mmol/L. Blood and cerebrospinal fluid cultures were negative. Liver and kidney functions were normal. Chest X-rays and abdominal CT scans showed no abnormalities. The head MRI revealed nodular enhancement in the sellar region, enhanced pia mater in the right pontine cistern and fourth ventricle, and communicating hydrocephalus (Fig. 1). The patient had a history of exposure to tuberculosis. Based on positive results from an interferon-gamma (IFN-γ) release assay, the patient was treated with rifampicin, isoniazid, and ethambutol, which resulted in minimal improvement.

**Fig. 2:**
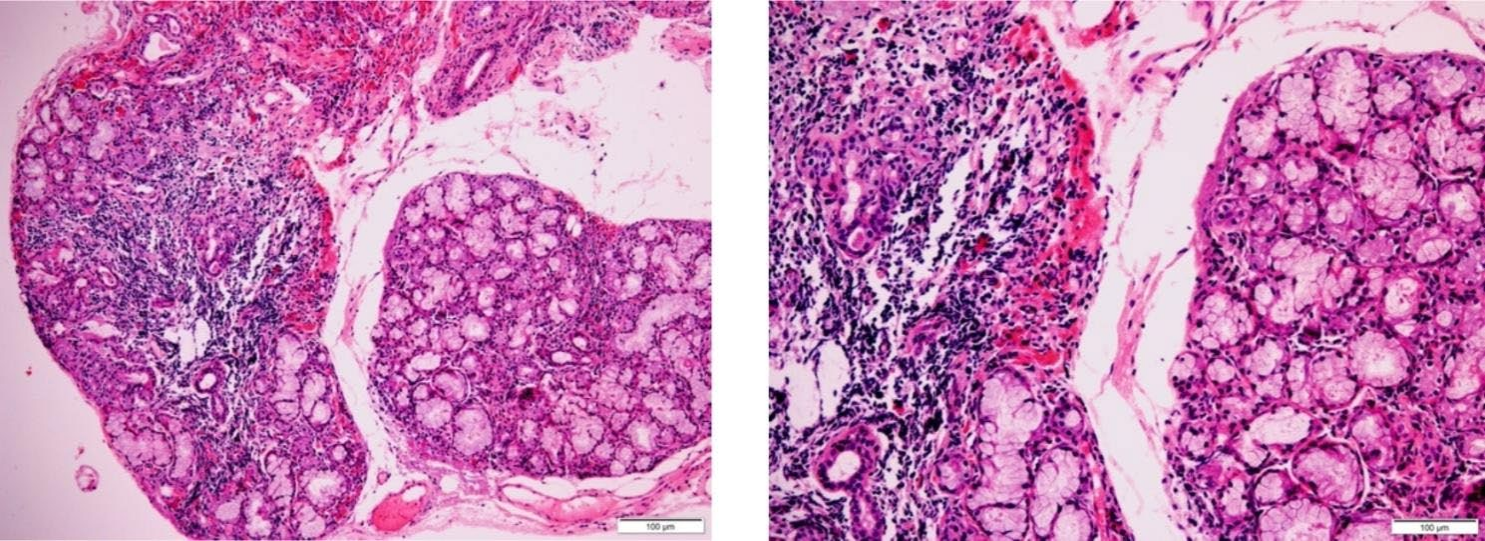
Labial gland biopsy shows the presence of lymphoid lesions surrounding the ducts. The picture on the left: At least one lesion was detected within a 4 mm^2^ tissue Sect. (10$\times$); The picture on the right: There are more than 50 lymphocytes infiltrating the lesion (20$\times$)

Additionally, the patient’s serum showed elevated levels of anti-Sjögren’s-syndrome-related antigen A and anti-Sjögren’s-syndrome-related antigen B (SSB) autoantibodies, erythrocyte sedimentation rate (ESR), cyclic citrullinated peptide (CCP) antibodies, perinuclear antineutrophil cytoplasmic antibodies (p-ANCA), and rheumatoid factor, indicating a possible diagnosis of SS. Therefore, we performed Schirmer’s test on the patient’s eyes (right eye:10 mm, left eye: 3 mm), tear film breakup time (right eye: 3 s, left eye: 1 s), and a corneal staining test (negative results). Moreover,

we conducted a biopsy of the patient’s labial gland which reveals focal lymphocytic sialadenitis with lymphocyte count $\ge$1 focus (50 lymphocytes/4mm²) (Fig. 2). Subsequently, the patient received intravenous dexamethasone for 2 weeks, and after one month of treatment, the patient’s CSF white blood cell count returned to normal levels, while protein levels remained elevated and glucose levels remained low. The patient’s headache symptoms improved, and she was discharged while continuing to take oral prednisone and anti-tubercular agents. It is worth mentioning that the patient resides in the southern region of Guangdong, China, where mice are frequently observed near the vicinity of her living quarters.

**Fig. 3:**
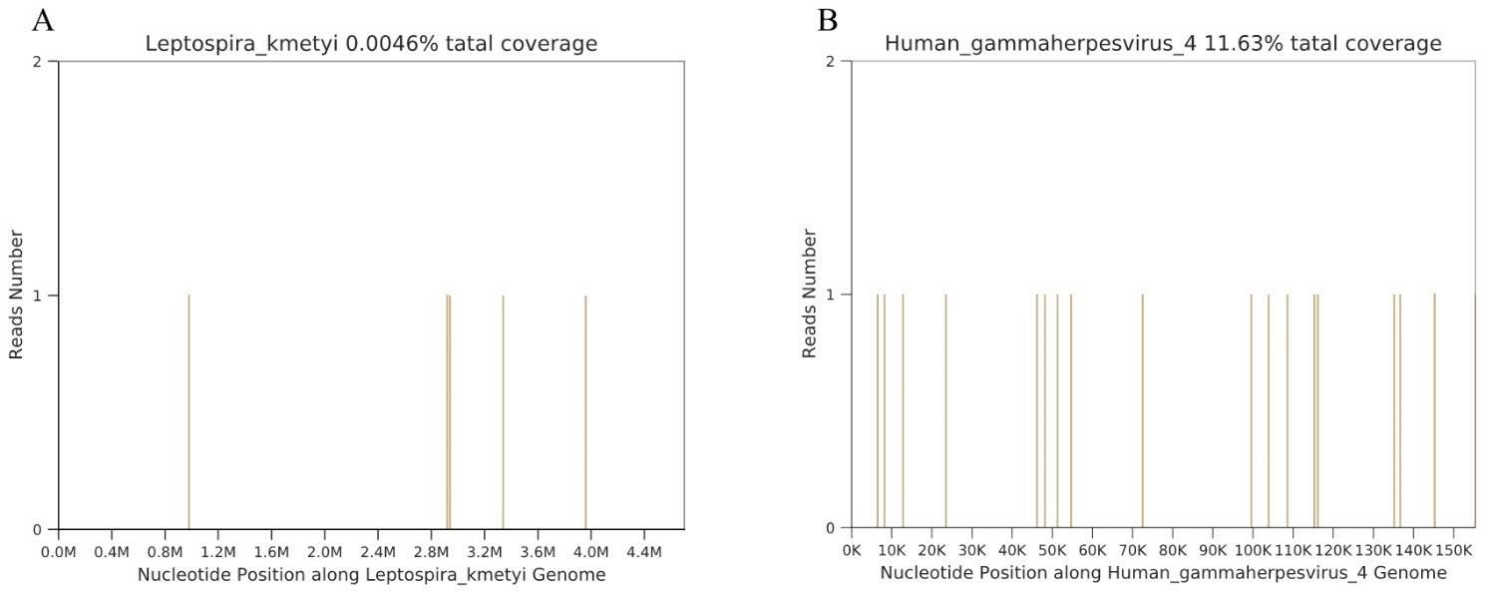
Report of PMseq of CSF (distribution of the locations in the pathogen genome). PMseq, pathogenic microorganism DNA/ RNA high-throughput genetic sequencing; CSF, cerebrospinal fluid; The “M” on the x-coordinate means the location of the genome. **A:***Leptospira*, **B:** Human-$\gamma$ herpes virus 4 (EBV).

After the patient was discharged for 2 months, abdominal pain and headache recurred in November 2019. Gastrointestinal endoscopy showed no significant abnormalities. The patient was treated with anxiolytic drugs and continued to take methylprednisolone, anti-tubercular drugs. Eight months later, in August 2020, the patient was re-admitted due to recurring headache and dizziness. Upon obtaining written consent from the patient and their family members, further pathogenic microorganism DNA/RNA high-throughput sequencing (HTS) detection of CSF revealed the presence of the *Leptospira.* HTS entails the extensive sequencing of nucleic acid molecules within specimens, subsequently aligning them with reference databases to achieve the analysis, detection, and tracing of infectious diseases [6]. However, we did not conduct further antibody testing on CSF and serum. Other pathogens such as Epstein-Barr virus (EBV) were also detected (Fig. 3). The therapy plan was modified to include penicillin (600IU/day, IV) and ceftriaxone (2 g/day, IV). Despite these interventions for one week, the patient’s CSF protein remained elevated (2960 mg/L), prompting the administration of immunosuppressive agents, such as azathioprine and hydroxychloroquine. Following negative results on IFN-γ release assay, tuberculosis drugs were discontinued. Follow-up of patients in December 2022, the cranial MRI exhibited persistent leptomeningeal enhancement and hydrocephalus. Accordingly, the patient’s immunosuppressive regimen was empirically adjusted to mycophenolate mofetil (MMF). CSF analysis indicated a marked reduction in white blood cells (9 cells/mm^3^) and protein (1729 mg/L) levels, with glucose (2.57 mmol/L) returning to normal limits.

**Fig. 4:**
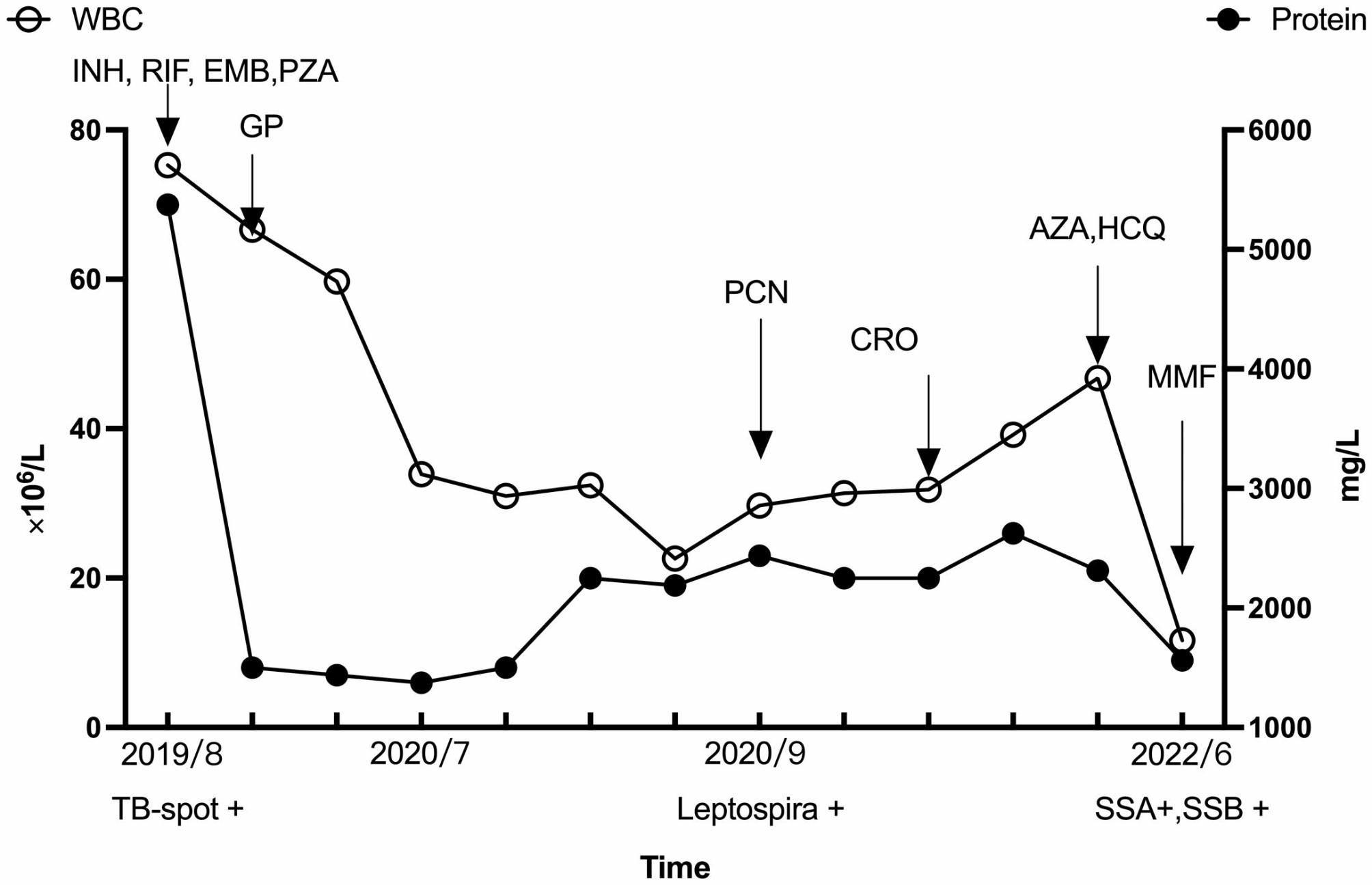
The schematic shows the levels of white blood cells (hollow circles) and protein (solid circles) in cerebrospinal fluid between August 2019 and June 2022. The downward arrows on the chart indicate the start of drug treatments. INH: isoniazid; RIF: rifampicin; EMB: ethambutol; PZA: pyrazinamide; GP: glucocorticoids; PCN: penicillin; CRO: ceftriaxone; AZA,azathioprine; HCQ, hydroxychloroquine; MMF, mycophenolate mofetil; TB-SPOT+,positive interferon-γ release assay; SSA + SSB+, positive anti-Ro and anti-La antibodies

The patient underwent a total of 13 lumbar punctures with examination of CSF over a period of 3 years, with intracranial pressure maintained at 160–180 mmHg and the CSF mainly clear and pale yellow. The patient’s CSF white blood cell count showed exhibited a gradual decline. Antituberculous drugs were administered for a duration of 2 years, while the patient received long-term corticosteroid therapy. While the CSF protein levels significantly decreased, they failed to recover to normal levels. In the later stages of treatment, immunosuppressants such as azathioprine, hydroxychloroquine, and sulfadiazine were added, but there was no significant effect on reducing CSF protein. Ultimately, the use of MMF and Penicillin was attempted, resulting in the patient’s CSF protein levels returning to normal (Fig. 4).

## Discussion and conclusions

Leptospirosis, characterized by clinical manifestations, primarily divides into various forms: anicteric leptospirosis and icteric leptospirosis [7]. This case pertains to a non-icteric meningitis variant. The diagnosis of leptospirosis typically necessitates a triangulation of clinical symptoms, epidemiological history, and laboratory examinations. In the current case, the patient’s enduring experience of cephalgia and cervical rigidity spanning three years implied a potential incidence of meningitis, with a suspected history of exposure to rodent excreta further arousing suspicion of leptospirosis. However, given the absence of Guangdong as a typical epidemic region for leptospirosis, coupled with the consistency and complexity of the symptoms, the conclusive diagnosis relies heavily upon the outcomes of laboratory investigations. In this particular instance, employing the methodology of HTS, we managed to successfully detect the DNA of *Leptospira interrogans* within the CSF sample of the patient. Additionally, the detection of EBV4 DNA in CSF raises the possibility of primary EBV infection or reactivation. Although central nervous system involvement in EBV infection predominantly occurs in immunocompromised individuals [8], it is important to acknowledge that EBV infection may also be a trigger for ADs [9].

Leptospira-specific antibody detection employs the microscopic agglutination test (MAT) and enzyme linked immu- nosorbent assay (ELISA) methods. MAT demonstrates high specificity but reduced sensitivity, potentially yielding false negatives in early-stage infections. Conversely, IgM ELISA exhibits heightened sensitivity in early infection but is susceptible to false positives due to potential cross-reactivity with other pathogens [10]. Neuroleptospirosis primarily manifests as aseptic meningitis, while additional neurological presentations encompass encephalitis, intracranial hemorrhage, cerebellitis, and myelitis [11]. Leptospirosis constitutes an infrequent etiology of aseptic meningitis, contributing to approximately 5–13% of such cases. The initial symptoms in patients commonly involve the frontal lobe headache, which is a non-specific manifestation of leptospirosis.

The ambiguity of symptoms and the lack of specificity in CSF examination results render the differentiation from other infectious meningitides challenging, leading to a delay in diagnosing leptospirosis. Moreover, this case received an extended course of anti-tuberculosis treatment with minimal effectiveness, underlining the vital role of molecular diagnostics in the differential diagnosis of meningitis. HTS technology, also known as NGS, holds numerous advantages compared to traditional pathogen culture methods [12]. Firstly, it facilitates the detection of uncultivable organisms, such as difficult-to-culture viruses or microbes [13]. Secondly, in comparison to traditional molecular biology techniques such as polymerase chain reaction (PCR), HTS demonstrates superior sensitivity and specificity, enabling the identification of low-concentration pathogen nucleic acids and even offering effective detection for rare pathogens [14]. Additionally, this technology can uncover novel, previously unidentified pathogens [15]. By comparing and analyzing unknown sequences, it provides fresh insights for disease diagnosis and etiological research.

More research indicates a potential correlation between pathogenic infections and the onset and progression of ADs. Microbes can initiate, amplify, or conversely, extinguish the immune response [16]. Infections can both stimulate and inhibit ADs. A retrospective clinical study has suggested a connection between leptospirosis and the subsequent risk of ADs, especially SS [5]. However, the underlying mechanisms bridging the two remain enigmatic. Animal studies have corroborated that the immune response triggered by *Leptospira* leads to pulmonary hemorrhage [17]. The antibodies against LruA and LruB, produced in response to *Leptospira*, have been implicated in the onset of uveitis [18]. Furthermore, through a molecular mimicry mechanism, leptospirosis may also induce antiphospholipid syndrome [19]. Some case reports hypothesize that leptospirosis serves as an aberrant trigger factor for systemic lupus erythematosus [20]. *Leptospira* immunoglobulin-like (Lig) proteins, through their interactions with intercellular adhesion molecules-1 and integrins, adhere to host cells, thereby assisting in evasion from the innate immune mechanisms [21].

SS is an ADs wherein the immune system aberrantly targets secretory glands, such as the salivary and lacrimal glands. This case features a co-diagnosis of leptospirosis and SS, the coexistence of which could be elucidated by several plausible mechanisms. Firstly, both leptospirosis and SS involve immune system anomalies. The inflammation and immune response induced by *Leptospira* might, to some extent, influence the progression of SS. Research has revealed that false-positive serological IgG ELISA results for leptospirosis in patients with ADs may arise from the presence of anti-nuclear antibodies, anti-phospholipid antibodies, and anti-cytoplasmic antibodies [22]. Immune system irregularities and immunosuppressive treatments also heighten the risk of infection [23]. Secondly, In SS, the function of CD4 + regulatory T lymphocytes declines, whereas the cellular immunity mediated by leptospirosis leads to a reduction in CD4 + lymphocyte count. Both factors collectively contribute to immune dysfunction.

Finally, the shared inflammatory processes elicited by leptospirosis and SS may interact and amplify each other, leading to disease variations. *Leptospira* and cholinergic autoantibodies from primary SS can both inhibit Na/K-ATPase, mediating the production of inflammatory mediators [24, 25]. Despite these hypotheses and speculations, the current research regarding the relationship between leptospirosis and ADs remains inadequate. Due to the uniqueness of this case, it is not feasible to directly conclude a definitive link between leptospirosis and ADs. Additionally, given the patient’s preexisting autoimmune condition prior to the onset of leptospirosis, ADs may serve as one of the potential triggers for leptospirosis. Notably, various factors, such as the patient’s prolonged use of immunosuppressive medications and a history of indirect contact with rodent excreta, contribute to the susceptibility to leptospirosis. Therefore,

further experimental and clinical studies are necessary to explore potential mechanisms and substantiate this correlation.

The utilization of antibiotics and immunomodulatory drugs proved beneficial in this patient’s treatment. Considering the concurrent diagnosis of ADs and leptospirosis, therapeutic drug monitoring can optimize the treatment efficacy. For instance, the pharmacokinetics of MMF can be leveraged, using quantitative analyses of its blood concentration to guide individualized dosing [26]. Although this case lacks drug monitoring, continual surveillance was undertaken on the patient’s CSF and serum as indicators for evaluating meningitis and immune function.

This case underscores the necessity of recognizing atypical manifestations of leptospirosis, fortifying the collection of medical history and epidemiological data, and enhancing physicians’ comprehension and diagnostic capabilities for leptospirosis. Clinicians should consider the possible complication of leptospiral meningitis when patients with Sjogren’s syndrome show neurological symptoms. It is crucial to augment doctors’ alertness and the importance of broad pathogen diagnostics for timely and accurate patient diagnosis and treatment. When it comes to the assessment of potential pathogens, performing a simple PCR is adequate rather going for expensive methods like HTS.

Treating leptospiral meningitis against the backdrop of an ADs presents a plethora of challenges and complexities, necessitating multidisciplinary involvement, including infectious disease specialists, rheumatologists, and neurologists, to devise treatment strategies and balance infection control with the management of autoimmune disease.

### Electronic supplementary material

Below is the link to the electronic supplementary material.

Supplementary Material 1

## Data Availability

The datasets generated during the current study are not publicly available due to privacy concerns but are available from the corresponding author on reasonable request.
