# Supplementary material for: Leptospirosis-associated meningitis in a patient with sjögren’s syndrome: a case report
Source: BMC Infect Dis. 2023 Nov 9;23:778. doi: 10.1186/s12879-023-08794-9 (PMC10636908; doi:10.1186/s12879-023-08794-9)
Supplement: Supplementary file 1 — Supplementary Material 1 [file 12879_2023_8794_MOESM1_ESM.pdf]

CONSENT FORM FOR CASE REPORT

Respected patients:

Thank you for your willingness to share your case for publication. We require your informed consent before your case report can be published. Please read the following content carefully, and sign this informed consent form if you agree.

Patient information:

Name: (Kunxiu Liu)

Age: (49 years old)

Gender: (female)

Diagnosis: (Leptospirosis meningitis, Sjogren's syndrome)

Informed consent:
I agree to the use of my case information for academic and scientific purposes and to the publication of my case report in a relevant medical or academic journal.

I understand and agree to the following:

1. Privacy protection: I understand that my personal identity and privacy will be kept strictly confidential, and no personally identifiable information will appear in the case report. My real name and personal information will be replaced or omitted to ensure privacy protection.
2. Academic purposes: My case reports will only be used for academic and scientific purposes, and may be used for medical education, disease research, formulation of clinical guidelines, and promotion of knowledge in the medical community.
3. Rights reserved: I understand that once my case report is published in a journal, copyright will vest in that journal. However, I still reserve the right to use my case information for other academic and scientific purposes.
4. Peer Review: Peer review may be required before my case report is published. This means that other medical experts will review my case report, evaluate it and suggest revisions.
5. Right to withdraw: I have the right to withdraw my consent before my case report is published. Once my case report has been published, it cannot be retracted.
6. Risks: Although we will do our best to protect my privacy and personal information, there may be certain risks in the process of information transmission and storage, including but not limited to information leakage. I am willing to take these risks.
7. Other uses: My case information will only be used for case report publication, and will not be used for other commercial or profit-making purposes.

Statement of consent:
I have read the above carefully and have sufficient knowledge to publish my case report. I agree to the use of my case information for academic and research purposes and understand that it may be subject to peer review. I confirm that my personal privacy and rights will be protected.

Patient Signature: 刘昆秀 (KunXiuLiu)

date: 23.5.20

Physician's signature: Yifan Zhang YongZheng

date: 2023.5.18
